# Supplementary material for: Macroscopic and microscopic study on floral biology and pollination of Cinnamomum verum Blume (Sri Lankan)
Source: PLoS One. 2023 Feb 2;18(2):e0271938. doi: 10.1371/journal.pone.0271938 (PMC9894414; doi:10.1371/journal.pone.0271938)
Supplement: S1 Fig — (A) Lateral view f Stamen Fourth whorl (S4) (B) First-whorl stamen at anthesis (with open stoma flaps); mature pollen grains at the opened pollen sac and the filament with fewer trichomes (C) Valvular dehiscence from the first whole stamen, Mature pollen grains distinct in opened pollen sac. (DOCX) [file pone.0271938.s001.docx]

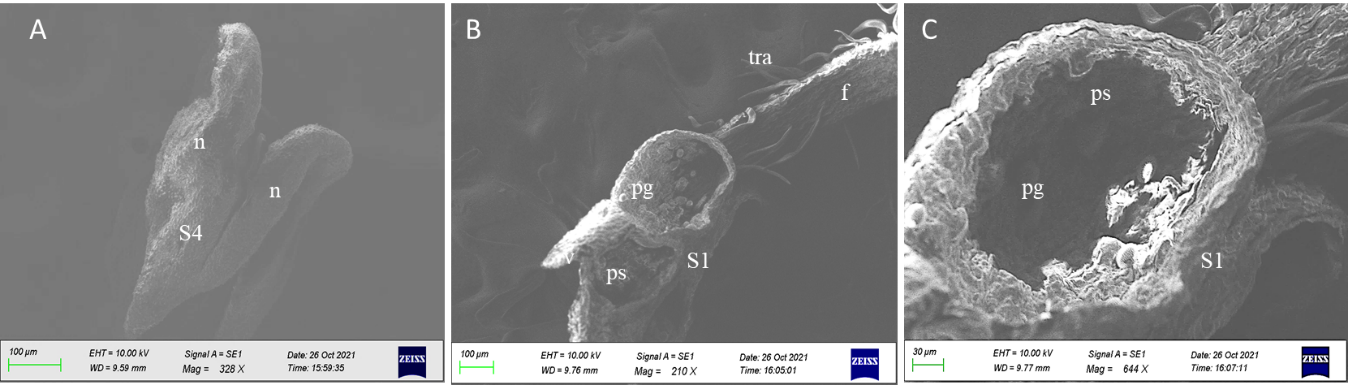


**Supplementary Fig. 1: SEM images of dehisced stamens in male flower** (A) Lateral view f Stamen Fourth whorl (S4) (B) First-whorl stamen at anthesis (with open stoma flaps); mature pollen grains at the opened pollen sac and the filament with fewer trichomes (C) Valvular dehiscence from the first whole stamen, Mature pollen grains distinct in opened pollen sac
