# Supplementary material for: Macroscopic and microscopic study on floral biology and pollination of Cinnamomum verum Blume (Sri Lankan)
Source: PLoS One. 2023 Feb 2;18(2):e0271938. doi: 10.1371/journal.pone.0271938 (PMC9894414; doi:10.1371/journal.pone.0271938)
Supplement: S2 Fig — Progression of overlapping percentage in Sri Gemunu of both female and male flowers during the overlapping period in peak and off peak season, with temperature, and humidity a-f G1,G2,G3,G4,G5,G6. (DOCX) [file pone.0271938.s002.docx]

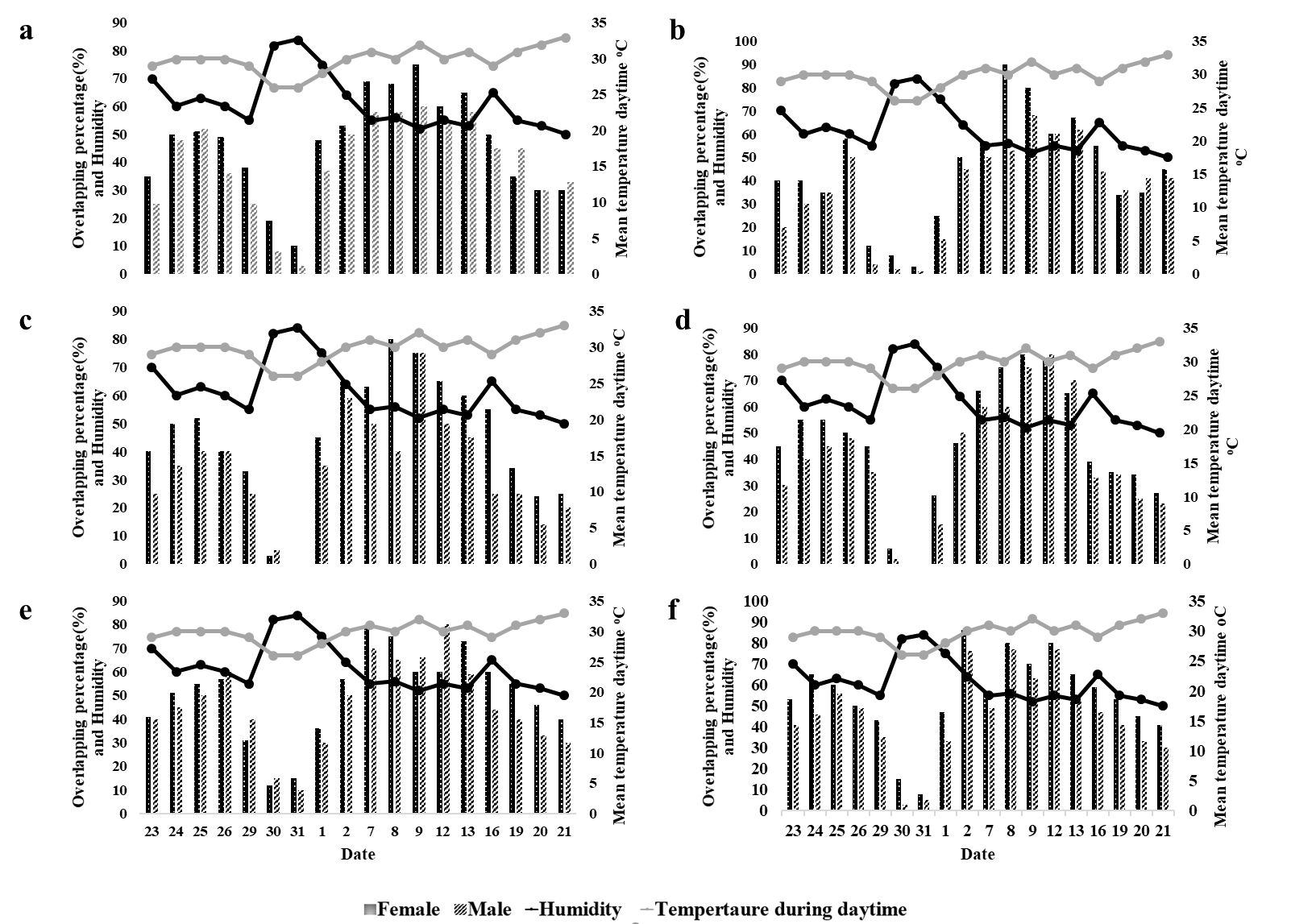


**Supplementary Fig. 2:** Progression of overlapping percentage in *Sri Gemunu* of both female and male flowers during the overlapping period in peak and off peak season, with temperature, and humidity a-f G1,G2,G3,G4,G5,G6
