# Supplementary material for: Macroscopic and microscopic study on floral biology and pollination of Cinnamomum verum Blume (Sri Lankan)
Source: PLoS One. 2023 Feb 2;18(2):e0271938. doi: 10.1371/journal.pone.0271938 (PMC9894414; doi:10.1371/journal.pone.0271938)
Supplement: S3 Fig — Progression of overlapping percentage in Sri Wijaya of both female and male flowers during the overlapping period in peak and off peak season, with temperature, and humidity a-f W1,W2,W3,W4,W5,W6. (DOCX) [file pone.0271938.s003.docx]

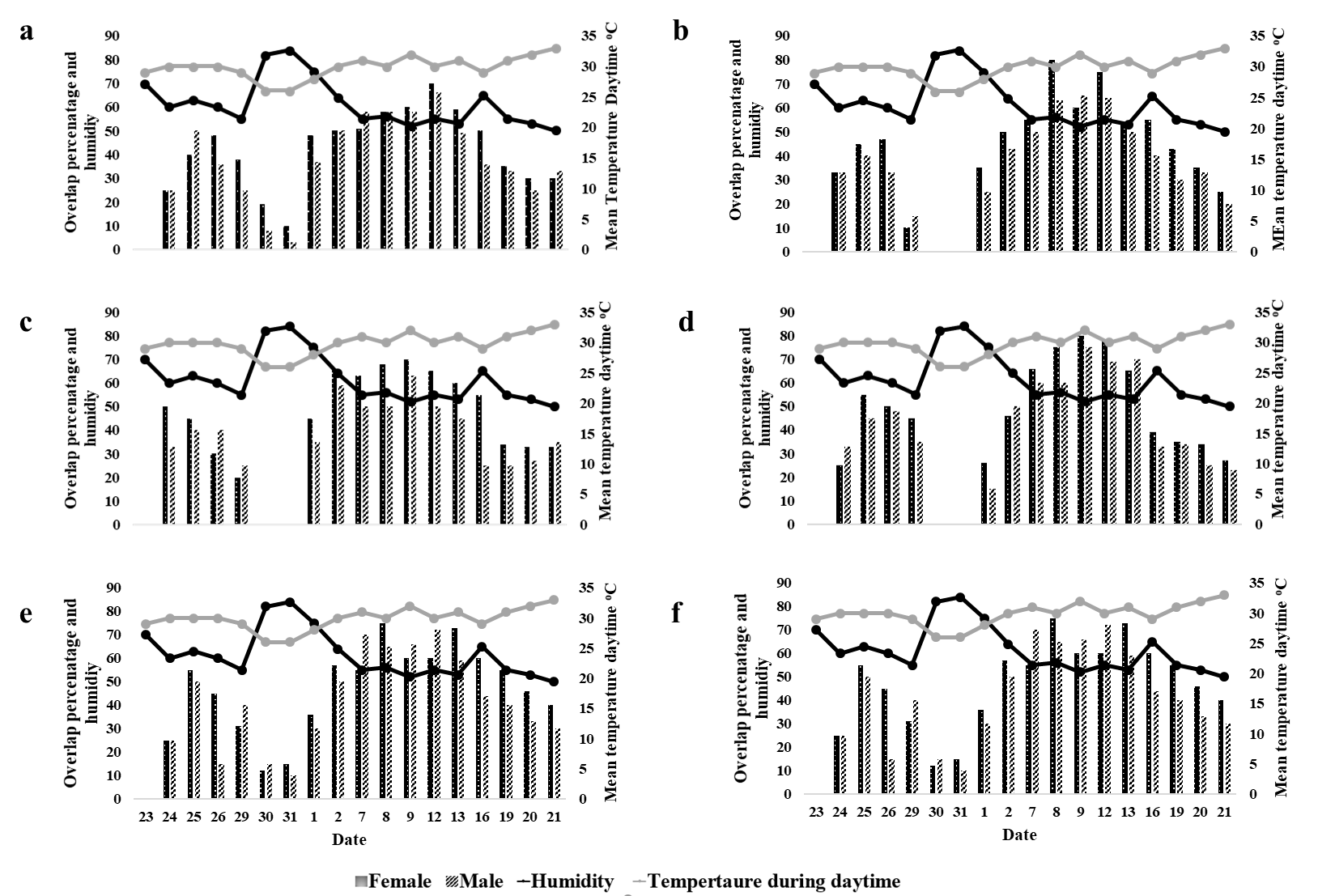


**Supplementary Fig. 3:** Progression of overlapping percentage in *Sri Wijaya* of both female and male flowers during the overlapping period in peak and off peak season, with temperature, and humidity

a-f W1,W2,W3,W4,W5,W6
