# Supplementary material for: Macroscopic and microscopic study on floral biology and pollination of Cinnamomum verum Blume (Sri Lankan)
Source: PLoS One. 2023 Feb 2;18(2):e0271938. doi: 10.1371/journal.pone.0271938 (PMC9894414; doi:10.1371/journal.pone.0271938)
Supplement: S4 Fig — (A) Lateral view of the fresh female stigma soon after opening (B) Dorsal ventral view of the fresh female stigma (sg) soon after opening (C) Stigmatic surface with elongated stigmatic papillary cells (pc) in ribs-like structures, providing higher surface area for pollen adhesion (D,E) Secretory vesicles (sv) appeared on the stigma after the stigma is receptive, the secretory vesicles are circular lobes-like structures, arranged all over the papillae cell layer (F) Pollen grains deposited on the female stigma, pollen merged on the stigmatic surface in the female flower (G) Pollen grains submerged on the stigmatic surface for the pollen tube growth, after pollination (H) Magnified Functional male stigmatic surface with multiple pollen deposited, all those pollen grains merged into the stigmatic surface, the pollen grains are shrunk and shrivelled. (I) Pollen grain submerged on the stigmatic surface for pollen growth detected in male stigma (J) Filaments observed in the style in the male flower stigma (K) Central groove observed in the style after pollination (L) The stigma has a central depression forming a short stigmatic cleft in the pollinated stigma, feather-like papillary cells are distinct. (DOCX) [file pone.0271938.s004.docx]

**
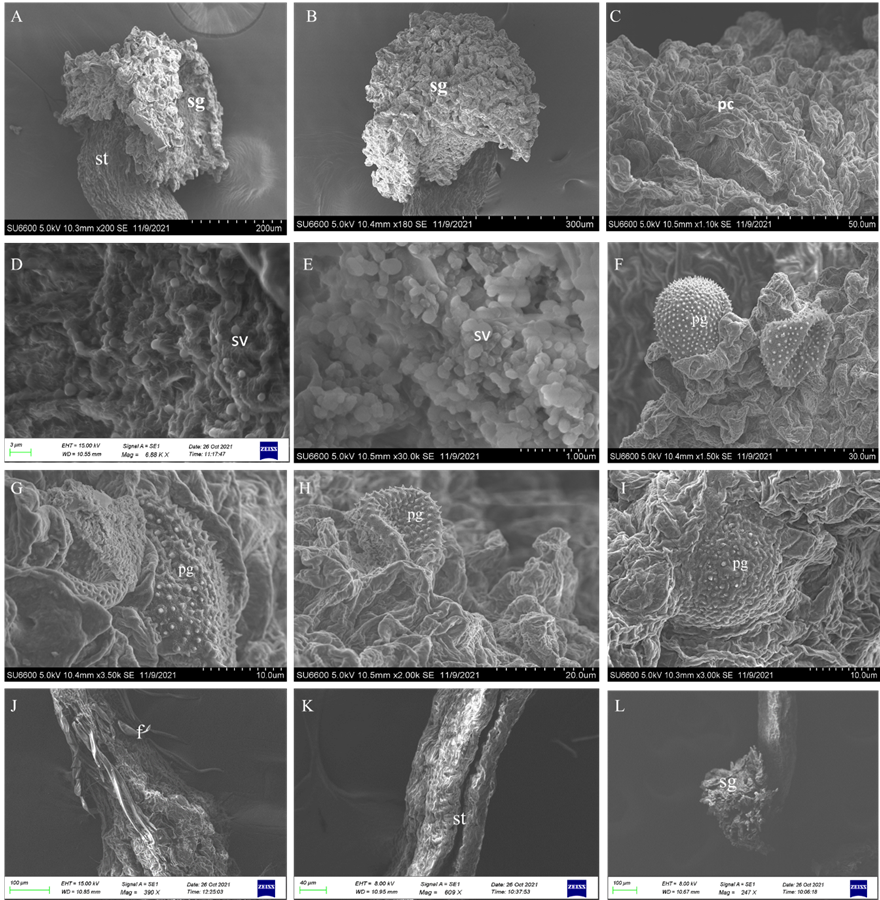
**

**Supplementary Fig. 4: Stigma morphology variation in *Sri Wijaya* during the floral cycle (A)** Lateral view of the fresh female stigma soon after opening (B) Dorsal ventral view of the fresh female stigma (sg) soon after opening (C) Stigmatic surface with elongated stigmatic papillary cells (pc) in ribs-like structures, providing higher surface area for pollen adhesion (D,E) Secretary vesicles (sv) appeared on the stigma after the stigma being receptive, the secretary vesicles are circular lobes-like structures, arranged all over the papillae cell layer (F) Pollen grains deposited on the female stigma, pollen merged on the stigmatic surface in the female flower (G) Pollen grains submerged on the stigmatic surface for the pollen tube growth, after pollination (H) Magnified Functional male stigmatic surface with multiple pollen deposited, all those pollen grains merged into the stigmatic surface, the pollen grains are shrunk and shriveled. (I) Pollen grain submerged on the stigmatic surface for pollen growth detected in male stigma (J) Filaments observed in the style in the male flower stigma (K) Central groove observed in the style after pollination (L) The stigma has a central depression forming a short stigmatic cleft in the pollinated stigma, feather-like papillary cells are distinct
