# Supplementary material for: Macroscopic and microscopic study on floral biology and pollination of Cinnamomum verum Blume (Sri Lankan)
Source: PLoS One. 2023 Feb 2;18(2):e0271938. doi: 10.1371/journal.pone.0271938 (PMC9894414; doi:10.1371/journal.pone.0271938)
Supplement: S1 Table — (DOCX) [file pone.0271938.s005.docx]

Summary of the data analysis

| **Study conducted** | **Measurements taken/Factors considered** | **Experiment details** | **Analysis conducted and Software used** | |
| --- | --- | --- | --- | --- |
| ***Flower and inflorescence morphology*** | Flower length (mm) |  | Image capture software - AmScope V/ 3.7.2776 and Olympus Cell Sens Standard V 1.16Image J software | Fisher’s least significance difference (LSD) using SAS studio version 3.8 |
|  | Flower diameter (mm) |  |  |  |
|  | Pedicle length (mm) |  |  |  |
|  | Inflorescence length (mm) |  |  |  |
|  | Stigma diameter (mm) |  |  |  |
|  | Style length (mm) |  |  |  |
|  | Petal length (mm) |  |  |  |
|  | Petal width (mm) |  |  |  |
|  | First and second whorl anther length (mm) |  |  |  |
|  | Flowers per inflorescence |  |  |  |
| ***Floral phenology*** | Temperature |  |  | |
|  | Humidity |  |  |  |
|  |  | Data collected period: December to the middle of February The trees were randomly selected |  |  |
|  | Total flowers open in female stage | Sri Gemunu: G1,G2,G3,G4,G5,G6; Sri Wijaya: W1,W2,W3,W4,W5,W6 |  |  |
|  | Total flowers open in male stage | Sri Gemunu: G1,G2,G3,G4,G5,G6; Sri Wijaya: W1,W2,W3,W4,W5,W6 |  |  |
| ***Floral Behaviour: Overlapping Period*** | Overlapping percentage of the selected plants were calculated: |  | Spearman’s rank correlation | Analyse-it Standard Version 2.30 |
|  | The period where both female stage and male stage flowers visible |  | Regression analysis to identify the overlapping between average humidity and average temperature at maximum, minimum and mean levels | Minitab version 17 (State College, PA: Minitab, Inc) |
|  |  |  | Synchrony ratio of female and male flowers | 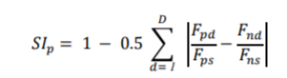 |
| ***Changes of Stigma Surface at Different Flowering*** | Qualitative measures were observed using Scanning electron microscopy images |  |  |  |
|  | Samples for the analysis were collected during the period of December - January |  |  |  |
|  | The collected stigmas were from the flowers that were pollinated via insects during the overlapping period. |  |  |  |
|  | The stigmatic surface was observed to identify the structural changes |  |  |  |
| ***Histological evidence for self-incompatibility*** | Qualitative measures were observed using scanning electron microscopy |  |  |  |
|  | Stigmas that were self-pollinated within same tree and within same cultivar was observed |  |  |  |
|  | The samples were taken on December 2021 |  |  |  |
