# Supplementary material for: Macroscopic and microscopic study on floral biology and pollination of Cinnamomum verum Blume (Sri Lankan)
Source: PLoS One. 2023 Feb 2;18(2):e0271938. doi: 10.1371/journal.pone.0271938 (PMC9894414; doi:10.1371/journal.pone.0271938)
Supplement: Attachment — Submitted filename: Response to reviewers.docx [file pone.0271938.s007.docx]

Journal Requirements:

When submitting your revision, we need you to address these additional requirements.

1. Please ensure that your manuscript meets PLOS ONE's style requirements, including those for file naming. The PLOS ONE style templates can be found at

https://journals.plos.org/plosone/s/file?id=wjVg/PLOSOne_formatting_sample_main_body.pdf and

https://journals.plos.org/plosone/s/file?id=ba62/PLOSOne_formatting_sample_title_authors_affiliations.pdf

2. Thank you for stating the following financial disclosure:

"The Ministry of Primary Industries and Social Empowerment through the National Science Foundation of Sri Lanka under the special Cinnamon project – Grant No: NSF SP/CIN/2016/01. "

Please state what role the funders took in the study. If the funders had no role, please state: "The funders had no role in study design, data collection and analysis, decision to publish, or preparation of the manuscript."

If this statement is not correct you must amend it as needed.

Please include this amended Role of Funder statement in your cover letter; we will change the online submission form on your behalf.

3. Thank you for stating the following in the Funding Section of your manuscript:

"Funded by the Ministry of Primary Industries and Social Empowerment through the National Science Foundation of Sri Lanka under the special Cinnamon project – Grant No: NSF SP/CIN/2016/01."

We note that you have provided funding information that is not currently declared in your Funding Statement. However, funding information should not appear in the Acknowledgments section or other areas of your manuscript. We will only publish funding information present in the Funding Statement section of the online submission form.

Please remove any funding-related text from the manuscript and let us know how you would like to update your Funding Statement. Currently, your Funding Statement reads as follows:

"The Ministry of Primary Industries and Social Empowerment through the National Science Foundation of Sri Lanka under the special Cinnamon project – Grant No: NSF SP/CIN/2016/01. "

Please include your amended statements within your cover letter; we will change the online submission form on your behalf.

Additional Editor Comments:

As the manuscript has been precisely reviewed by the experts in the disciplines and after thoroughly going through their reports it is cleared that there are some shorts comes which need to be address before publishing it so the decision is recorded as a Major Revision. Enclosed please find the reviewers’ report on your paper. I suggest to the authors a complete typographical and grammatical revision. Most of the references used to seem a little old to me, I suggest that the authors update and insert new references, to corroborate the assertions presented by the authors. Statistical section is very weak and need major attention in this section. So, according to the recommendations made by the valuable reviewers, the manuscript needs comprehensive major revisions.

[Note: HTML markup is below. Please do not edit.]

Reviewers' comments:

Reviewer's Responses to Questions

Comments to the Author

Reviewer #1: The manuscript presented is of good importance and will be benefit for the scientific community.

Here are my comments and suggestions:

1. The abstract should be rewrite and authors should present clearly this section to capture their result and how this will be useful.

The abstract was revised following the instructions of the reviewer.

2. No proper flow in the introduction and too difficult for the readers to understand the authors.

The introduction was revised in order to make it easy for the reader to understand

3. Material and method need adjustment: I will invite authors to summarize in a table format all the data capture, code use, period of collection and nature of the data (qualitative/quantitative).

The data was included in the table (after the figure legends) as suggested by the reviewer.

4. The analysis section very weak and authors need to address this points (which software was used for the analysis, which model was used, how the factors were considered/radom/fixed) in addition to this authors should add the regression analysis to display how the weather parameters impact on the flower both males and females.

The suggested analysis methodology was included in the methodology section line number 215-230.

The regression analysis suggested was included in table 3 and as well as Fig 8.

5. Did authors test the pollen viability before conduction hand pollination. if not how sure authors relies on the current results.

Thank you for the suggestion. However, since we have sure results from scanning electron microscopy and also from the fruiting

6. In the results section, some sections look more like discussion. please move those sentences into the discussion section.

Results section was adjusted. Sections similar to discussion was moved to discussion section.

7. Please use correlation analysis considering only the quantitative variables to see the relationship among the variables (you can use as well the the variety type as factor see this link <https://r-graph-gallery.com/199-correlation-matrix-with-ggally.html>

Thank you for your suggestion. The correlation analysis was carried out for quantitative variables using Minitab vr: 7 software. The above mentioned software was not used for the analysis. Included as Figure 11.

8. In table 1, add the result of the LSD for mean separation.

The Values are added with means ± SD of all the female and male stage flowers in Sri Gemunu and Sri Wijaya (n) =10 replicates, Means denoted by the different letters within the same column are significantly different at P<0.05 (Table 1)

9. The discussion section needs to be adjusted, only discuss the major discovery and how this will be used to improve the production. Also check for english typo across the document

Discussion was adjusted as instructed by the reviewer. English typos were also corrected.

Reviewer #2: The submitted manuscript does not have good theoretical bases on plant reproductive biology as it should have. The presented objectives are very unclear, the methods are not adequate, and the results does not support the conclusions.

Thank you for the review. The objectives, methods, data analysis and results were revised as suggested.

1. The objetive presented here is very vague.

The objective in the introduction was rewritten

2. The atributes analysed do not characterize the resource accessibility. Besides, why do the authors need to characterize such resource? Are they flowers resources for pollinators? it was not explained.

The explanation for the analysis was explained in line 123-124 as reviewer suggested.

3. Which data was tested and analysed?

The data analysis section was rewritten with broad explanation number 215-230.
